# Perspectives and Experiences of Public Safety Personnel Engaged in a Peer-Led Workplace Reintegration Program Post Critical Incident or Operational Stress Injury: A Qualitative Thematic Analysis

**DOI:** 10.3390/ijerph21070949

**Published:** 2024-07-19

**Authors:** Chelsea Jones, Shaylee Spencer, Elly O’Greysik, Lorraine Smith-MacDonald, Katherine S. Bright, Amy J. Beck, R. Nicholas Carleton, Lisa Burback, Andrew Greenshaw, Yanbo Zhang, Phillip R. Sevigny, Jake Hayward, Bo Cao, Suzette Brémault-Phillips

**Affiliations:** 1 Heroes in Mind, Advocacy and Research Consortium (HiMARC), Faculty of Rehabilitation Medicine, University of Alberta, Edmonton, AB T6G 2R3, Canada; shaylee1@ualberta.ca (S.S.); ellyogreysik@ualberta.ca (E.O.); smithmac@ualberta.ca (L.S.-M.); kbright@ualberta.ca (K.S.B.); ajbeck@ualberta.ca (A.J.B.); psevigny@ualberta.ca (P.R.S.); suzette2@ualberta.ca (S.B.-P.); 2 St. Stephen’s College, University of Alberta, Edmonton, AB T6G 2R3, Canada; 3 School of Nursing and Midwifery, Mount Royal University, Calgary, AB T3E 6K6, Canada; 4 Department of Psychology, University of Regina, Regina, SK S4S 0A2, Canada; nick.carleton@uregina.ca; 5 Department of Psychiatry, Faculty of Medicine and Dentistry, University of Alberta, Edmonton, AB T6G 2R3, Canada; burback@ualberta.ca (L.B.); andy.greenshaw@ualberta.ca (A.G.); yanbo9@ualberta.ca (Y.Z.); bcao2@ualberta.ca (B.C.); 6 Department of Educational Psychology, Faculty of Education, University of Alberta, Edmonton, AB T6G 2R3, Canada; 7 Department of Emergency Medicine, Faculty of Medicine and Dentistry, University of Alberta, Edmonton, AB T6G 2R3, Canada; jhayward@ualberta.ca; 8 Department of Occupational Therapy, Faculty of Rehabilitation Medicine, University of Alberta, Edmonton, AB T6G 2R3, Canada

**Keywords:** mental health, first responders, public safety personnel, peer support, return to work, workplace reintegration, operational stress injury, critical incident

## Abstract

Introduction: Public safety personnel (PSP) experience operational stress injuries (OSIs), which can put them at increased risk of experiencing mental health and functional challenges. Such challenges can result in PSP needing to take time away from the workplace. An unsuccessful workplace reintegration process may contribute to further personal challenges for PSP and their families as well as staffing shortages that adversely affect PSP organizations. The Canadian Workplace Reintegration Program (RP) has seen a global scale and spread in recent years. However, there remains a lack of evidence-based literature on this topic and the RP specifically. The current qualitative study was designed to explore the perspectives of PSP who had engaged in a Workplace RP due to experiencing a potentially psychologically injurious event or OSI. Methods: A qualitative thematic analysis analyzed interview data from 26 PSP who completed the RP. The researchers identified five themes: (1) the impact of stigma on service engagement; (2) the importance of short-term critical incident (STCI) program; (3) strengths of RP; (4) barriers and areas of improvement for the RP; and (5) support outside the RP. Discussion: Preliminary results were favorable, but further research is needed to address the effectiveness, efficacy, and utility of the RP. Conclusion: By addressing workplace reintegration through innovation and research, future initiatives and RP iterations can provide the best possible service and support to PSP and their communities.

## 1. Introduction

### 1.1. Background

Public safety personnel (PSP) experience operational, personal, and organizational stressors that put them at heightened risk of developing mental health challenges compared to civilian populations [1]. A broad and evolving “term to describe the people who ensure the safety and security of the public” [2], PSP includes “border services officers, serving Canadian Armed Forces members and Veterans, correctional service and parole officers, firefighters (career and volunteer), Indigenous emergency managers, operational intelligence personnel, paramedics, police officers, public safety communicators (e.g., 911 dispatchers), and search and rescue personnel” [3]. One Canadian study found that 36.7% of municipal police, 34.1% of firefighters, 50.2% of Royal Canadian Mounted Police (RCMP), and 49.1% of paramedical staff surveyed screened positive for one or more mental health conditions including but not limited to posttraumatic stress disorder (PTSD) [1].

Operational stress injuries (OSIs) is a term “often used by PSP to describe mental health problems that may result from performing their assigned duties” [3]. OSIs are not listed as a diagnosis in the Diagnostic and Statistical Manual of Mental Disorders (DSM-5-TR) [4]. The term can encompass a range of mental health conditions, including PTSD, major depressive disorder, and various mood-, anxiety-, trauma-, and substance use-related disorders [3] as well as moral injury and burnout. OSI symptoms may result in diminished cognitive functioning, increased social isolation, difficulty with interpersonal relationships, and increased workplace absenteeism [5,6]. Functional impairments from OSIs contribute to challenges with returning to work in a present, meaningful, and engaged manner [7]. An unsuccessful return-to-work process can exacerbate financial and mental health distress for the PSP member and their family as well as contribute to staffing shortages that can have adverse consequences for PSP organizations and the communities they serve. Although the economic burden of OSIs among Canadian PSP is unknown [8], estimates of productivity losses for mental health disorders among the Canadian population due to factors like absenteeism range from 16.6 to 21 billion annually [9,10,11].

Despite the importance of successful return-to-work experiences and processes, the published literature on workplace reintegration after clinical rehabilitation to address OSIs is limited. Initiatives to address gaps between clinical care and functional workplace tasks are beginning to emerge through grassroots efforts by various PSP organizations. Many PSP organizations have recognized peer support as a component of an overall work reintegration strategy that may help PSP return to work following injury. Formalized peer support refers to a variety of mental health supports and resources implemented via programs or services and offered by a peer who provides social, emotional, spiritual, and instrumental support to promote well-being and recovery [3,12]. Historically, PSP peer support has been based on a shared lived experience, such as similar employment or exposure to potentially psychologically injurious events (PPIEs) [3,13]. Based on a review of the literature on initiatives designed to support PSP in their workplace reintegration after incidents, Sutton and Polaschek (2022) [14] identified the incorporation of peer support, focusing on stress and well-being outcomes and education, across all types of studies but noted “a dearth of research into the influence of critical incident interventions on work performance, attitudes or experiences” (pp. 732). In formal peer-support initiatives, the knowledge and expertise possessed by the peer supporter through their lived experience is often supplemented with additional training and mental health knowledge [3,15,16].

Peer-support programs are difficult to evaluate in an implementation science or research context. This is because there does not exist standardization among these programs, making examination of the safety, effectiveness, efficacy, and impact of such initiatives challenging [16]. The training and experience of peer supporters, level of formalization and structure, accessibility (i.e., virtual versus in-person), and the mandates or goals of peer-support initiatives vary widely across organizations, professions, and jurisdictions [10,17,18]. To improve the evaluation of the effectiveness of peer-support programs and crisis-focused psychological intervention programs, the current literature recommends further standardization of research and peer-support programs, rigorous methodological research designs, and research engagement of independent, qualified, and established researchers [10,17,18].

### 1.2. A Peer-Led Workplace Reintegration Program

The Edmonton Police Service (EPS) identified a need to assist police officers who were off work following some PPIE exposures designated as “critical incidents”. Operational definitions of critical incidents can vary among PSP organizations and professions but often include officer-involved shootings and events where serious injury or death of an individual has occurred. Workplace reintegration efforts were introduced in 2009 as a response to this issue, by providing officers with a peer-led program to guide them through their critical incidents and regain confidence in operational skills [19]. The EPS Reintegration Program (RP) has evolved and spread to several other PSP organizations and professions across Canada, the United States, and New Zealand [7,20]. RPs may include elements specific to each PSP organization and profession, but the goal of the program remains to assist PSP with returning to work as soon as possible following a PPIE, illness, or injury while diminishing the potential for long-term psychological injury [19].

The RP incorporates short-term critical incident (STCI) and long-term RP streams [19]. Detailed descriptions of the RP have previously been published in detail [20]. The STCI stream is a proactive psychological program [10] offered to PSP immediately after a critical incident to assist with rebuilding confidence in skills and providing support in hopes to mitigate the potential future impact of an OSI on workplace reintegration and engagement. This stream may also incorporate mandatory time away from work and engage clearance and short-term intervention by a medical or mental health professional prior to full return to duty. Both streams of the RP include elements of relationship building, reorientation to equipment related to their role, skill building, and exposure to common workplace scenarios. PSP enrolled in the RP are guided by a trained peer RP facilitator who assists with exposure to unique stressors that the PSP may experience in their role [19]. Peer support offered by RPs is complementary to clinical interventions but outside the scope of what officers receive from their healthcare professionals [19]. Peer-support duties within the RP expand beyond the scope of basic peer support and into a formalized program of meaningful assistance, for which the RP facilitators receive specialized training [15,16]. The previous literature on RPs has emphasized the importance of having the right fit for peers in the role as facilitators, including a positive reputation and respect by peers within the organization, to facilitate buy-in and reduce stigma [7,16].

Research on initiatives targeting PSP after PPIEs similar to the RP have recently been published. A Canadian literature review reported limited evidence supporting post-exposure peer support and crisis-focused psychological interventions, which may include critical-incident stress debriefing, stress management, peer support, psychological first aid, and trauma risk management in isolation or combination, in mitigating PTSIs among PSP [16]. A literature review from New Zealand noted promising preliminary findings for peer-led tertiary interventions, indicating that further evaluation and research of these types of programs, including the RP, is warranted [14]. Published studies on the RP itself are limited but include work describing program stakeholder perspectives [7], introducing internally collected program statistics [19], and clarifying the outcomes of the RP facilitator training [15,16]. Research addressing whether this specific RP is effective, efficacious, or safe from an evidence-based perspective is yet to be completed. Initiatives such as the RP must be evaluated to clarify current effectiveness, support continuous improvement, and protect investment returns.

### 1.3. Research Question and Study Purpose

This qualitative study aimed to explore experiences and perspectives of PSPs who had engaged in the workplace RP designed by EPS after experiencing a PPIE or an OSI. This study aimed to address the following research questions: (1) What are the experiences of PSP engaged in the RP after a PPIE or OSI? (2) Do PSP engaged in the RP perceive it as effective in assisting in their return-to-work process? The study results can provide valuable information on participant perspectives regarding the impact of the RP on PSP returning to duty. Based on previous research, PSP participants were expected to view the RP as a favorable element of the workplace reintegration process [7,20].

## 2. Materials and Methods

This qualitative study used an overarching community-engaged and pragmatic research approach [21,22] with a thematic analysis to better understand the perspectives of PSP regarding the workplace RP within Alberta, Canada. Ethical approval was received from the Research Ethics Board at the University of Alberta (Pro00118357).

### 2.1. Participants and Recruitment

Potential participants for the current study included PSP who had participated in an RP program within their organization. Participating organizations included EPS, Alberta Health Services Emergency Medical Services, St. Albert Fire Services, and the RCMP. Study participants were recruited through purposeful sampling. Potential participants were asked by their RP facilitator at their initial contact if they would be interested in participating in the study. Study team members contacted potential participants who consented to be contacted. After screening for eligibility, willing PSP provided written consent to participate in the study. PSP were eligible if they had taken time off work due to experiencing a PPIE identified as a critical incident or an OSI. As a result, the participants had engaged or were about to engage in the STCI or long-term stream of the workplace RP within their respective organizations. Participants were not eligible if they had engaged in the RP due to time off of work for a medical illness, musculoskeletal injury, or maternity leave that did not directly include a PPIE or psychological challenges related to the workplace. Recruitment took place from May 2022 to September 2023.

### 2.2. Data Collection

Quantitative and qualitative data were collected between May 2022 and December 2023. Consent and demographic questionnaires were administered via REDCap (Research Electronic Data Capture), which is a secure, web-based software platform [23]. The research team designed a deductive semi-structured interview script based on the research questions and after consultation with key partners of the RP. Steps used to develop the guide included (1) considering which event(s) illustrate phenomena of interest, (2) ordering questions to optimize flow, and (3) refining the schedule through a series of review and piloting [24]. Based on the iterative process and in line with suggested qualitative research methodology for the development of interview scripts for thematic analysis [25], six topic areas were included in the interview: (1) events that led to being off work; (2) level and type of support; (3) steps in the reintegration process; (4) relationships with key partners and organizations; (5) perceived strengths, facilitators, and barriers of the RP; and (6) perceived effectiveness and efficacy of the RP (Table 1).

Participants were asked to participate in an interview at three months post initiation of their involvement with either the STCI or long-term stream of the RP. Individual interviews (*n* = 26) lasting approximately 60 min were conducted and recorded over a secure Zoom video conferencing link according to the existing literature and guidelines for qualitative data collection [26]. Data collection continued until information power was reached [25]. Information power considers study focus, sample specificity, established theory, quality of dialogue, and analysis strategy, which would ideally also correlate with data saturation [27].

### 2.3. Data Analyses

Quantitative data were captured and analyzed descriptively using Microsoft Excel software. Qualitative data in the form of audio or video-recorded interviews were professionally transcribed. Data were anonymized at this step by assigning a participant number to each transcript. The transcripts were then thematically analyzed deductively and inductively following an iterative process [26]. Initial codes were developed through an inductive process by identifying both manifest and latent themes that presented in the data. The semi-structured interview script was deductive and included specific questions about the RP. Manifest content emerged, including content related to logistic aspects of the RP, that was analyzed at face value.

There were two researchers (S.S. and E.O.) who independently conducted open coding of the transcribed data for each interview, after which arms-length researchers (C.J. and S.B.-P.) reviewed and provided feedback on the codes. Axial coding and the development of preliminary themes followed, with disagreements being resolved through discussion.

Trustworthiness [27] was considered throughout and facilitated through the clear problem statement, development of a relevant research question, and an a priori research method. Appropriate and thoughtful qualitative approaches were used to address the research question with flexibility [28,29,30]. Additionally, critical reflexivity was considered throughout data analyses, and discussions regarding researcher positionality, perspectives, relationships, and biases were engaged at multiple points throughout the study [28].

Once the final themes were determined, key quotes were isolated to illustrate the themes, and the final presentation of the thematic analysis was prepared. The Standard for Reporting Qualitative Research was used to guide the reporting process [31].

### 2.4. Triangulation

The overarching mixed-methods research study used method and data triangulation of multiple qualitative and quantitative approaches and sources of which have [7] and will be reported in future peer-reviewed manuscripts. A concurrent parallel approach following a data transformation model was used in this larger initiative’s final data analysis process to converge the data for comparing and contrasting the quantitative results with qualitative findings [32].

## 3. Results

### 3.1. Demographics

The demographic details of study participants (*n* = 26) are presented in Table 2 below. Study participants were an average age of 35.08 (SD = 7.95) years and belonged to one of Alberta’s four participating PSP organizations. Levels of experience within their respective fields varied, with the average years in their profession being 9.85 (SD = 6.45).

### 3.2. Qualitative Thematic Analysis Results

The thematic analysis revealed a breadth of information on the experience of the participants who engaged in the RP. The participants experienced numerous PPIEs and other workplace stressors throughout their career, all of which was conveyed through their stories and anecdotes. Participants were acutely aware of the potential for greater mental unwellness with increased exposures to PPIEs. Some participants had previously engaged in the RP after past PPIEs. Other participants had not had the opportunity for participation in an RP until present, as their past PPIEs had occurred before the installation of the RP within their organization. A few participants noted they had participated in the RP multiple times to date. Overall, the participants generally perceived the RP as positively impacting their workplace reintegration.

**Figure 1:**
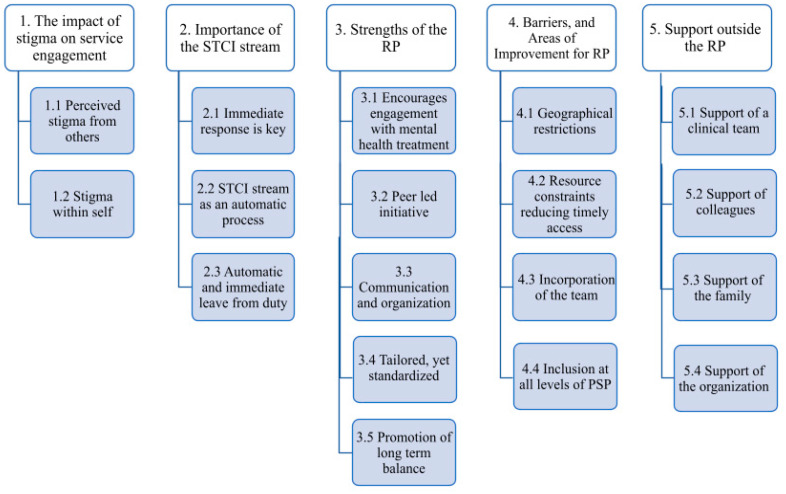
Qualitative Analysis Results.

The thematic analysis yielded five main themes: (1) the impact of stigma on service engagement; (2) importance of the STCI program; (3) strengths of RP; (4) barriers and areas of improvement for the RP; and (5) support outside the RP (Figure 1). The themes are summarized below, supported by detailed descriptions and supporting quotes.

#### 3.2.1. The Impact of Stigma on Service Engagement

Stigma was identified by participants as an important factor that affected decision-making regarding taking time off work, engaging in mental health services, and participating in other formal and informal peer support, including the RP. Participants reported experiencing stigma from colleagues and the organization in the workplace and within PSP themselves due to internalized pressure to perform and a strong professional identity. Stigma contributed to feelings of shame or guilt when affected by an OSI. Stigma from others or self was noted to potentially contribute to PSP staying in the workplace longer than is indicated with an OSI, avoiding seeking clinical treatment, and potentially attempting to navigate workplace reintegration without a formalized program such as the RP.

Despite stigma remaining a factor that may affect PSP engagement in services designed to assist them, many participants noted that mental health stigma has decreased and continues to decrease in the workplace and PSP professions. Improvements were largely attributed to numerous initiatives within PSP organizations and their professions designed to increase mental health knowledge, normalize discussions about mental health, improve recognition of the signs and symptoms of OSI, and encourage seeking help when needed (Table 3).

#### 3.2.2. The Importance of the STCI Stream

Participants who participated in the STCI stream of the RP endorsed its importance to the work RP after experiencing a PPIE in the workplace. Implementing the STCI stream was described as providing a prompt and supportive response for PSP to get directions on reintegration, peer support, and mandatory time away from work. Many participants felt that integrating the STCI stream into mandatory processes was a strength of the RP. The STCI stream was found to be a helpful process for those who engaged in it, positively impacting their personal and professional lives. Some participants noted that they were likely to voluntarily engage in the STCI stream as needed in the future.

It is important to note that not all of the PSP organizations had an STCI stream. A few participants reported that they wished to advocate for PSP organizations whose RPs did not include a STCI stream because they believed so strongly in the benefits of this approach and process (Table 4).

#### 3.2.3. Strengths of the RP

The participants identified perceived strengths of both the STCI and long-term streams of the RP. These included (a) encouraging clinical mental health engagement; (b) peer involvement; (c) communication and organization by the RP team; (d) a standardized program with the flexibility to adjust to individual needs; and (e) promotion of long-term health and wellness. The RP implementation created a community and minimized the impacts of social isolation. Participants conveyed that the RP assisted in their workplace reintegration and was a welcomed component of their recovery journey (Table 5).

#### 3.2.4. Barriers and Areas of Improvement for RP

The barriers and areas of improvement cited by participants varied among PSP organizations, as each had its own unique jurisdictions, demands, resourcing, and slight variations in program design. Many participants across the PSP organizations identified geographical restrictions as a barrier to participation in the RP. Some also noted that factors such as resourcing restricted timely access to the RP after a critical incident. Additionally, some participants conveyed that incorporation of their team members would be a benefit to the reintegration process. Participants also reported that the RP is viewed by others within the organizations as an initiative for those on the front lines of PSP work, and PSP in more administrative or supervisory roles may feel less welcome. Refer to Table 6 for further detail.

#### 3.2.5. Support outside the RP

The RP was described as a source of support during the return-to-work process; nevertheless, participants reported other support as important for their workplace reintegration, such as clinical support from a healthcare team through formal OSI treatments. Additionally, support, or a lack of it, from colleagues, family, the profession, and the organization were identified as potentially affecting the success of the process by either adversely contributing to or mitigating stressors during workplace reintegration (Table 7).

## 4. Discussion

The current qualitative study was designed to explore the experiences and perspectives of PSP who had engaged in a workplace RP due to experiencing a PPIE, identified as a critical incident, or OSI. The current study is the first to address the perceived effectiveness and utility of a specific workplace RP, demonstrating favorable results. The results of this study successfully provided insight into the following research questions: (1) What are the experiences of PSP engaged in the RP after a PPIE or OSI? (2) Do PSP engaged in the RP perceive it as effective in assisting in their return-to-work process? Overall, PSP felt favourably towards the RP and reported that they found it was a valuable component of their return-to-work journey after a PPIE or OSI.

Most participants identified as men (77%), which is consistent with the gender estimates among policing professions in Canada [33]. Many participants were in their early to mid-career (*n* = 22), and over half (*n* = 15) had participated in the STCI stream of the RP opposed to the long-term stream (*n* = 11). Having entered the program immediately after a PPIE may be, in part, why most of the participants had already returned to work at full capacity. As data collection commenced at three months post entry into the RP, it remains possible that some participants may still be affected by an OSI in the future.

Through the qualitative thematic analysis, five themes were developed. Of note, the themes shared some similarities to the recent qualitative literature regarding the return to duty of Canadian PSP as well as discussions of peer support and the current state of programs and initiatives that aim to mitigate the effects of PPIEs on PSP.

Stigma remains an influential factor in the decision making of PSP regarding seeking mental health support, taking time off from work, and engaging formal and informal services [32], which may include the RP. Participants noted internal and perceived external stigma as a barrier to acknowledging OSIs and seeking treatment, which is consistent with the recent literature [34,35,36,37]. An overall reduction in stigma and increase in mental health literacy is being observed among PSP populations [14,15,16,35], which the data from this study corroborated. Reducing stigma, therefore, may facilitate workplace reintegration [35,37].

The STCI stream of the RP is a peer-led, tertiary intervention [14] that is used by some of the PSP organizations after a PPIE exposure that meets the organization’s criteria for being a critical incident. The current results suggested participants felt the STCI was a particularly important part of the RP. Important elements included automatic participation post PPIE, mandated leave from duty after this event, and the immediate engagement of the RP facilitators. Further research is warranted on the impact of the mandatory nature of workplace reintegration initiatives and time off work after exposure to PPIEs.

Participants reported several strengths of the RP. First, the RP reportedly encouraged participants to engage with mental health treatment and was often responsible for providing resources for the PSP member to connect with healthcare professionals. Second, participants identified the peer-led aspect as an integral strength of the workplace reintegration process. This result is consistent with the previous literature establishing the benefits of both formal and informal peer supports related to reducing mental health stigma [36,37,38,39,40] and facilitating PSP workplace reintegration [7,14,35]. Third, the ability to accommodate and tailor the RP approach within the standard format was a program strength. Pacing exposure activities and graduating the return to duties in a way that fostered choice and control within the individual was paramount to fostering trust with the RP facilitator as well as others within the PSP organization. An individualized and tailored approach to workplace reintegration has been recommended in the literature for civilian populations and is also emerging in the literature specific to PSP [16,35,37].

The importance of high-quality communication strategies as part of the reintegration process was emphasized as a contingency for the satisfaction and success of returning to work after illness or injury in multiple sectors. This was also an evident theme among study participants, which is consistent with the literature regarding workplace reintegration of PSP [16,35,37,41]. Workplace reintegration is a complex process that requires coordination of multiple parties, necessitating timely, clear, and genuine communication between the individual and others, with varying privacy and confidentiality requirements [39,41]. This need may be amplified when PSP are experiencing mental health symptoms or acute stress due to a critical incident [41]. Most participants felt the RP enhanced communication throughout their involvement.

Lastly, participants appreciated the focus on the balance of their professional and personal lives and emphasis on sustained wellness. Other studies of PSP engaged in workplace reintegration also cited aspirations of improving lifestyle behaviours, finding joy, and managing stress over the long term [35].

Barriers and areas of improvement related to the RP were also noted in the findings. Geographical restrictions and difficulties with adequate resourcing to meet demands were noted by participants, which is consistent with previous publications [7]. The incorporation of the team into the RP as well as encouraging a safe and welcome program for all ranks, including leadership, were ideas that emerged in the data as potentially strengthening the RP.

Support from outside the RP was observed as an important element of a successful workplace reintegration after a critical incident or OSI. Healthcare providers were noted as a support used by many of the participants. A recent Canadian study found that during the workplace reintegration process, PSP were most likely to access psychologists, occupational therapists [42], and family physicians when engaged with a workers compensation organization [35]. This study also noted that, due to the nature of PSP careers, it is advantageous for healthcare providers to be informed of the different roles and traumatic events that various PSP experience to improve engagement and outcomes with experienced healthcare providers being cited as a facilitator for return to work [35].

Colleagues within the profession and organization were noted to play an important role through informal peer support. Strong co-worker and supervisor support were observed as positive organizational factors that facilitate better mental health among PSP organizations [40]. Colleagues have been highlighted for the important part they play in the return-to-work processes [39,43], and it has been suggested that a more formalized role may facilitate improved reintegration experiences [44].

Family support was identified as an important factor in workplace reintegration, consistent with other literature [35,45]. Some PSP felt comfortable reaching out to their spouses or other family for support when needed. On the contrary, some participants did not feel comfortable speaking to their family, as they felt they would not understand the nuances and context of PSP work or may be burdened by the challenging nature of the work.

The importance of feeling supported by the PSP organization as a whole was important to participants. The RP was important for facilitating trust and connection with the organization. Connection with the workplace was previously noted as a facilitator of a more successful workplace reintegration for PSP [46,47]. Numerous publications have emphasized the importance of trust and buy-in with the organization in reintegration efforts for PSP [6,7,37,40,41].

### 4.1. Practice Recommendations

First, the continued facilitation of strong communication between the RP, individual PSP, and partner department, organizations, and healthcare teams appears particularly important [7]. Organizations using an RP without the STCI stream should consider trialing this piece of the RP. In the organizational context, RP teams should assess whether the intra-organizational messaging and marketing of the program fosters trust and feelings of inclusion for all levels of leadership, including PSP in supervisory and administrative positions.

RP delivery and training of RP facilitators should remain standardized to support implementation consistency across jurisdictions, professions, and organizations, thereby facilitating meaningful evaluations of effectiveness. Research and evaluation tools across RPs should also remain consistent. If research results regarding the RP are favourable, the use of effective implementation science and program evaluation approaches would best facilitate sustainable spread and scale with fidelity and quality control [16]. The use of validated pre–post surveys or outcome measures for PSP going through the RP would also provide useful and detailed information regarding the impact of the RP.

### 4.2. Research Recommendations

Research conducted by qualified arms-length research teams external to the PSP organizations is recommended for mitigation of bias and conflict of interest with programs like the RP and its partners [17]. Future research should incorporate constructs and conditions that may correlate with success in sustained return to work, such as measures of absenteeism, presenteeism, organizational injustice, work function, work performance, and perceived stigma as well as mental health knowledge and attitudes [8,15,18]. Studies on the organizational and cultural impact of the RP and a cost–benefit analysis would also be advantageous to assist in providing the best and most appropriate programming possible for PSP [8,9]. Studies that employ larger samples and quantitative methods are also needed to further address the question of efficacy, effectiveness, and utility of the program on constructs of return to work [17,18]. Both randomized and non-randomized studies are needed to inform further program modalities and delivery methods for PSP, which may include virtual means of service delivery [17,18]. Favourable research results would potentially pave the way for more widespread program adoption and integration that ensures risk management strategies and the maintenance of program fidelity [7,17].

The RP may also be a promising initiative to trial in other sectors where there is increased exposure to PPIEs, such as in healthcare settings [48]. Further research and exploration are still needed prior to the implementation, spread, and scale of the RP to other industries.

### 4.3. Limitations of Study

Limitations associated with the study include a convenience sample that drew on pre-existing relationships with a small number of PSP organizations. The reduced generalizability of the results must be noted, as differences in jurisdiction, government policy, internal processes, resourcing, needs of the community being served, and other factors may affect the implementation, delivery, and use of the RP. Results may vary in other provinces, states, or countries.

## 5. Conclusions

Research and initiatives that address clinical interventions for PSP with OSIs have increased over the past decade. The topic warrants investigation, investment, and advocacy; however, the policies, processes, and programs that facilitate successful workplace reintegration post clinical treatment must also be addressed through evidence-based evaluation and consideration. The results of this initial qualitative study demonstrated that participants view the RP as facilitating mental health support and assisting in their return to work. The STCI also emerged as being viewed as favorable by participants for assisting those after a critical incident. This study also highlighted additional variables outside the RP that PSP perceived as impacting workplace reintegration, including stigma and additional sources of support. Access to evidence-based RPs may facilitate retention of PSP and enable PSP to continue to be engaged, present, and healthy as they serve their communities.

The EPS RP is a grassroots effort specifically created to support the return-to-duty process of PSP. It has undergone global spread and scale even with the absence of published literature, which demonstrates the need and desire for a focus on workplace reintegration among PSP. Rigorous, independent, and outcome-based research is still needed to help meet the RP objectives. By addressing workplace reintegration through innovation and research, future program iterations can provide continuously improving service and support for PSP and their communities.

## Figures and Tables

**Table 1:** Examples of Questions in the Semi-Structured Interview Guide.

Interview Topic	Examples of Questions
Events that led to being off work	How long has it been since the critical or culminating incident(s) we are interviewing you about? Can you give us a short summary of the events that led to an OSI and time away from work?
2. Level and type of support	What was the pathway to engaging with professional help? What other supports did you have around you (colleagues, family, and friends)? Was your family offered support?
3. Steps in the reintegration process	What did reintegration involve for you? When you returned to work, did you feel ready?
4. Relationships with key partners and organizations	How did/do you feel about how you were treated by the organization (employer) after the OSI?
5. Perceived strengths and facilitators of the RP	What was the most useful/best part of the RP? Was there anything you found less helpful?
6. Perceived effectiveness and efficacy of the RP	Do you think that the RP will contribute or has contributed to missing more or less work since your OSI? Did the RP affect how much you engaged in available healthcare services and/or [Worker’s Compensation Organization] As a result of the RP, do you think you are more or less likely to stay in your profession/position for the long term?

**Table 2:** Sample Demographics.

Demographic Category (*n* = 26)	Participants *n* (%)
Gender	
Man or masculine	19 (73%)
Woman or feminine	6 (23%)
Transgender man, male, or masculine	0 (0%)
Transgender woman, female, or feminine	0 (0%)
Gender nonconforming, genderqueer, or gender questioning	0 (0%)
Two-spirit	0 (0%)
Not listed	0 (0%)
Prefer not to specify	1 (4%)
Age	
18–24	1 (4%)
25–34	11 (42%)
35–44	9 (35%)
45–54	2 (8%)
55–64	2 (8%)
65+	0 (0%)
Unknown	1 (4%)
Sex	
Female	6 (23%)
Male	20 (77%)
Intersex	0 (0%)
Prefer not to specify	0 (0%)
Ethnicity	
White	22 (85%)
South Asian (e.g., East Indian, Pakistani, Sri Lankan, etc.)	1 (4%)
Chinese	1 (4%)
Black	0 (0%)
Filipino	0 (0%)
Latin American	1 (4%)
Arab	0 (0%)
Southeast Asian (e.g., Vietnamese, Cambodian, Laotian, Thai, etc.)	0 (0%)
West Asian (e.g., Iranian, Afghan, etc.)	0 (0%)
Korean	0 (0%)
Japanese	0 (0%)
Indigenous, Metis, Inuit	3 (12%)
Other/Unknown	1 (4%)
Prefer not to say	0 (0%)
Education Level	
Some high school	0 (0%)
High School Diploma	4 (15%)
Vocational or technical college/College Diploma	8 (31%)
Some undergraduate	8 (31%)
Undergraduate degree	5 (19%)
Graduate degree	1 (4%)
Unknown	0 (0%)
Marital Status	
Married	15 (58%)
Common Law	3 (12%)
Separated	1 (4%)
Divorced	1 (4%)
Single	6 (23%)
Widow/Widower	0 (0%)
Prefer not to say	0 (0%)
Professional Role	
Municipal Police	12 (46%)
Federal Police	6 (23%)
Paramedic	7 (27%)
Firefighter	1 (4%)
Other	1 (4%)
PSP Organizations	
Edmonton Police Services	12 (46%)
Alberta Health Services	7 (27%)
Royal Canadian Mounted Police—K Division	6 (23%)
St. Albert Fire	1 (4%)
Number of Years in Profession	
<1	2 (8%)
2–5	5 (19%)
6–10	9 (35%)
11–15	6 (23%)
16–20	3 (12%)
21–25	0 (0%)
25+	1 (4%)
Unknown	0 (0%)
Current Work Status	
Full-time	20 (77%)
Part-time	0 (0%)
Causal	0 (0%)
Short-Term Disability	2 (8%)
Long-Term Disability	2 (8%)
Prefer not to say	1 (4%)
Unknown	1 (4%)
Which stream of the RP were/are you involved in?	
Short-Term Critical Incident	15 (58%)
Long-Term	11 (42%)

**Table 3:** The Impact of Stigma on Service Engagement.

Sub-Theme	Description and Supporting Quotes
Perceived Stigma from Others	Participants reported concerns over peer perceptions regarding their capacity to work if they admitted to having struggles and were witnessed going off work and engaging in services or initiatives such as the RP. Some participants conveyed that the widespread knowledge that an individual is experiencing an OSI may have negative career implications, impact career advancement, and compromise the trust of peers who they work alongside. Due to the perceived stigma from others within the organization, participants indicated that staying silent about their OSI was preferable and may reduce the likelihood of engaging in clinical or peer-led rehabilitative or workplace reintegration initiatives: *“We didn’t have a good track record of guys returning to work from our departments that actually returned successfully after a psychological injury… the fear of getting diagnosed with, like, PTSD diagnosis or anything with mental health. Earlier on, it wasn’t talked about, you didn’t want to bring it up. That being said, there’s been a lot of steps made and improvements made”.* P3 Many participants indicated that there has been improvement in recognizing the stigma associated with mental health concerns among co-workers and PSP organizations: *“…they talked about their mental health, like it was nothing like it was, you know, talking about the weather, they talk(ed) about their own experiences, like, you know, they’re walking their dog…seeing that was probably the most beneficial or useful thing because I saw like, …it’s, it’s really a culture of talking about it. Like there is no stigma, like the word nobody even said stigma. Like, it was really open. And so that was really eye opening for me and useful for me just knowing that that’s out there and that I can talk about it, and I don’t have to hide that”.* P20
Stigma within Self	Some participants related feeling embarrassed or defeated when experiencing mental health distress. They felt that they should be able to cope with the impact of critical incidents and workplace stress without assistance and had “failed” if events in the workplace had impacted them. Self-stigma was identified as a barrier to help-seeking by the participants: *“For me personally, I feel like I was admitting defeat. That was a big thing for me…because there’s still a huge stigma around mental health and policing and, and I felt like I was failing. So it was to go speak to a psychologist. For me it was a hard pill to swallow initially”.* P21 *“…the balls in your court, like, they can only do so much… if you’re too proud to accept the help, well, it’s 2023. And you’re a fool”.* P23

**Table 4:** The Importance of the STCI Stream of the RP.

Sub-Theme	Description and Supporting Quotes
Immediate Response is Key	Participants identified immediacy as a key positive aspect of the STCI stream. For PSP organizations using this stream, services and contact were to be enacted as soon as possible. Participants related that they may not have initially acknowledged that this process was necessary, but they saw the STCI stream’s value as they went through the program: *“I feel like it would be helpful, helpful for the member involved to have some one-on-one with someone. Right away”.* P12 Some participants were able to speak of past experiences of post-critical incident management prior to the implementation of the RP. They noted feelings of uncertainty, confusion, isolation, and fearfulness regarding taking time away from the workplace and returning to duty: *“I really wish it was offered when I said we were in a pursuit where the two (individuals) were killed. Because I remember I broke down then too, and I was put in an office and then given three days off to deal with it on my own”*. P18
STCI Stream as an Automatic Process	Most STCI stream participants suggested participation should be automatic and mandatory after a critical incident: *“Depending on the severity, of course, but it has to be mandatory somehow. But it’s not like, I don’t think you can do a cookie cutter. Like, every time you’re going to do this, you’re going to do that. But you have to meet with someone. And there has to be checks and balances to make sure you’re okay”.* P23 Most participants felt that they and their peers may not be able to recognize early indications their mental health has been impacted. The stigma associated with acknowledging mental health distress and asking for support was also reduced, as the decision was not left to the individual. Several participants indicated that they did not initially feel the critical event(s) had affected them and that their participation in the program was unnecessary but acknowledged the program’s importance retrospectively: *“I went through it, it felt very voluntary. I do think it should be mandatory. But just because it’s mandatory, it doesn’t mean that it’s, it’s this big, bad thing. Because when you do reintegration, it’s literally whatever you want it to be… you’re not locked into this certain program… they have that flexibility”*. P20 Participants acknowledged some initial challenges with insights into their mental health, potentially recognizing changes in personality and other mental health symptoms after some time had passed: *“I called my boss. And again, this is a TA [temporary acting supervisor] going off and she just said, ‘Hey, like trust the system. If they sidelined you, this is for a reason’. And I was like, ‘Okay, no, like, sounds good, I guess’. And then I got home and I told my wife. I said, ‘Can you believe in like, they fucking sidelined me?’. She’s like, ‘Thank God’. And I looked at her. She’s like, ‘You have been miserable. The last two days, you’re on edge… I was walking on eggshells around you’…”.* P23
Automatic and Immediate Leave from Duty	As part of the STCI stream of the RP, participants within the policing organizations (*n* = 15) were often mandated to take leave from duty for a period of approximately 14 to 30 days. Although participants may not have originally agreed with the mandated time off, they conveyed that this was helpful in retrospect: *“…if I wasn’t mandated, I would have gone back to work. The next set? Really? Yes, for sure. I would have been back there. And I wouldn’t have done any of it (the RP) and I want to deal with it. And it would have been, probably it would have, would have had a bad outcome for sure. So I think as much as I didn’t like it at the time, being told, you know, you can’t go to work. I think it should be depending on your level of involvement. I think it should be mandatory”.* P21 Participants felt the allotted time from work was adequate to allow them time to initiate the RP, complete administrative tasks, and connect with mental health professionals as needed. With mandated and automatic leave, there existed less pressure for police officers to return to work before they were ready. Those participants from the non-policing PSP organizations acknowledged that having mandatory time off after a critical incident may have been helpful for them. Despite the mandatory time off, some police officers still required additional time off later due to an OSI.

**Table 5:** Strengths of the RP.

Sub-Theme	Description and Supporting Quotes
Encourages Engagement with Mental Health Treatment	Participants identified that strong encouragement from RP facilitators to connect with mental health professionals assisted in reducing stigma. Some indicated that they might not have sought mental health assistance for treatment if it was not strongly recommended by the RP team. After the conclusion of the RP, some participants noted that they would continue to see a mental health professional to maintain mental wellness and address any future personal or work-related stressors: *“Just me seeing a psychologist. It’s been really helpful. Not only for this incident, but some other stuff that happened before that. I didn’t realize it was affecting me as well”.* P12 With the STCI stream in particular, knowledge that others involved in the same incident may also be seeking clinical services on the recommendation of the RP team contributed to the normalization of the mental health distress that can be attributed to a critical incident. Participants indicated the RP made accessing mental health assistance easier with resources such as a preferred provider list. Participants who experienced critical incidents prior to the implementation of the RP identified having to navigate an often confusing pathway for seeking mental health services: *“I would say that the reintegration program kind of engaged me in more services just based on the fact that, like, being so new to the organization, I don’t really know a lot about the benefits”.* P20
Peer-Led Initiative	The presence of peers as RP facilitators emerged as a notable highlight for PSP participants in the RP. Participants identified the importance of connecting with someone who knew the workplace demands, profession, culture, language, practices, and procedures associated with their role. The ability to share their experience with someone who comprehended the associated emotions, sensations, physical demands, and moral conundrums associated with critical incidents was noted to be invaluable. The participants reported that discussions with the RP facilitator enabled moments of clarity and understanding of workplace situations and events that the participants experienced negative thoughts about: *“And then just talking about with like, other first responders or police officers that have gone through similar events, and then now that helped guide people through the reintegration process. It is just nice to know that there’s, we can talk to them anytime we want, like reintegration never ends. They say if I ever have a problem, I can text [RP facilitators], and we can go for coffee, or we can work through whatever we need to work through”*. P22 *“…the most valuable part of the reintegration for me, was sitting there telling my story to two colleagues who have been in a similar situation…”.* P9 Being able to use specialized, profession-specific equipment in a safe setting for workplace exposures with a trusted peer was noted to improve confidence and skill building within the workplace environment.
Communication and Organization	The communication of the RP team and their standardized approach to organization of the process reportedly assisted PSP with navigating resources, services, and administrative tasks. Some participants noted that RP facilitators were quick to initiate connection with the participants once the referral for the RP was engaged. They noted that the RP team very clearly communicated their role and that of the RP while also providing clarity of the RP process and managing expectations: *“[RP facilitator] called me and was like, hey, like, this is what we do. This is what I do. Once we get your referral like, this is what can happen, you know, kind of explained all that to me”.* P20 A few participants indicated that communication between the RP and PSP could still be improved. They desired more direction on what to expect throughout their engagement with the RP team and more prompt communication. They felt the delay in communication was likely due to high demand on the RP team: *“I just think that there needs to be some sort of sit down and, and explain like, ‘Hey, this is kind of what you can expect, essentially, to come, come in the coming weeks, like you’re going to be getting a lot of people reaching out to you, you’re going to be getting a lot of emails that some of which you’re not going to open for months, because you don’t want to, you’re going to have to make sure that you keep on top of these specific tasks, like your payroll, your, your benefits, stuff like that’*”. P10 Participants reported developing a workplace reintegration plan that was well organized and clear through collaboration with the RP facilitator and other stakeholders, which could include healthcare providers, disability management departments, workers’ compensation organizations, and supervisors. This included the exposure activities as well as a graduated return-to-work schedule. Participants noted the choice and flexibility of the plan provided a sense of control within the standardized process. The clarity of the plan, expectations, and organization were cited by participants and major advantages of engaging in the RP: *“The reintegration team has the ability to see what calls are coming in…we would go to a low grade call. And then he would go in and talk to the crew who was inside the call and say, ‘Hey, we’ve got somebody reintegrating is it okay, if we, you know, come in on your call with you?’”.* P7 *“The reintegration (program) set the gradual return to work. Like I built the schedule with reintegration, which was really awesome*”. P10
Tailored, yet Standardized	Participants reported that the flexibility of the RP to be adapted based on the choice and needs of the participant was paramount to its success. Allowing the participant to provide input on the pace of reintegration and type of exposure activities allowed individuals to experience control of their return-to-work process. This approach was reported to reduce feelings of pressure to return to work too soon and allowed for advancement in the intensity, frequency, and time length of exposure activities as well as roles, shifts, and assignments in the workplace: *“I didn’t know much about what they did. So I started asking him about stuff they do. And like, you know, and he’s like, ‘Well, it’s always member driven. You guys decide what you want to do. We just help you get back’”.* P6 *“The very first day, we jumped out, one of the things that we did, we never actually left the ambulance at all we did was sit in the ambulance for almost three hours. Like that’s, that’s it. And it was like, it was just something where it’s just exposing, because I was having difficulty with that. And then and then it was doing things like let’s just go driving, because I was having very strong difficulty of sitting in the passenger seat because I was sitting in the passenger seat during my accident…and it was very much at my speed…they’re like, this is what we can do today. But if you’re not comfortable with this, we can focus on this instead, it was very much just slow and steady”.* P10
Promotion of Long-Term Balance	Participants highlighted the RP as promoting long-term health, wellness, and balance between their personal and professional life as a strength. Numerous participants noted that the RP encouraged self-care and sustainable mental health practices throughout the lifespan. Many participants noting they wish they would have had access to the RP and this knowledge earlier in their career. Some participants expressed a desire for an improved work/family/life balance extending into retirement: *“I want to be able to one day retire, enjoy my life, I don’t want to be riddled with these issues”.* P23 Participants identified having improved mental health knowledge because of the RP; focusing on self-care skills and strategies, triggers, mental health symptoms; and the impact of OSI on personal and professional relationships.: *“I’m… far more aware of my own mental health and, and far more aware of the like, for me what things are my warning signs you know, the just not wanting to go, being aware that one week of not wanting to go and just wanting to kind of batten down the hatches is okay. But when you start getting into, you know, month three of that something should have something should have, you know, pinged off and it just didn’t, I’m being really irritable, and that’s not me, you know, being insubordinate, which is not me. Those things to being aware of those things and being able to say, ‘Okay, I need it, I need some time to step back’”*. P7

**Table 6:** Barriers and Areas of Improvement for RP.

Sub-Theme	Description and Supporting Quotes
Geographical Restrictions	Some participants had the privilege of accessing the RP in the city in which they resided. Others reported having to commute to the RP facilitators or having to wait until RP facilitators were able to travel to their geographical area. Weather conditions, family needs, and work schedules were some of the factors that complicated travel for both RP facilitators and participants. For those who had to travel to attend the RP, higher levels of stress were reported due to being away from home and often having to travel to unfamiliar locations. The reduced access to the RP was especially problematic for those PSP who were located in isolated areas where both the RP and clinical mental health supports and services were difficult to access: *“…I have been going every two weeks for psychology appointments. So, and that’s me driving from (remote location) to (city center) every two weeks”.* P2
Resource Constraints Reducing Timely Access	Some participants identified that engaging with the RP closer to the time of the critical incident would have been beneficial to their long-term well-being. Multiple factors, such as the number of PSP involved, type of PPIE, availability of RP facilitators, and availability of resources to access remote geographical locations, impacted PSP wait times to engage in the RP. Increased time between the event and engaging in the RP had the potential to increase the length of time off work for those in the STCI stream of the RP, especially within organizations where the RP was a mandatory step prior to re-engaging in regular duties: *“There’s only two full timers…they just need like, yeah, they need more people [RP facilitators] for sure”.* P15 *“…there’s so many people reintegrating…there was also still people being reintegrated from officer involved shootings, like different ones…there were courses going on and stuff like that. So right, it was mostly just the range schedule that prolonged it”.* P17
Incorporation of the Team	Participants identified that the RP focuses on one-to-one interactions with RP facilitators. As teamwork, collaboration, loyalty, and partnership are integral characteristics of PSP roles, some participants noted that incorporation of other team members who may have been involved in the same critical incident may assist with reducing isolation and increasing both individual and team confidence: *“I missed the camaraderie with my squad. And just being around people who want to kind of understand the whole situation, what you’re going through”.* P22 Exposure activities and team-building initiatives, where appropriate and agreed upon by all participants, could be an innovative way to incorporate peer support and understanding: *“…one thing I noticed is, you’re isolated from your friends…from my squad alone, I think there was six of us on scene…they’re mandated to do reintegration as well…we’re really close as a squad. One thing that was never offered, and I brought it up to some guys after, is there was never any option presented for, like, group reintegration…or even, ‘We’re all gonna go sit down for lunch. Would you like your buddies to be there?’ Or, ‘You know what, you’re gonna sit around a table and discuss it…’ You spend more time with your squad than you do your family sometimes”.* P21
Inclusion of all levels of PSP	Some participants noted a gap in RP services based on rank and position within the PSP organization. They conveyed that PSP who are more advanced in their career, within leadership or administrative roles, and are removed from front-line work are still impacted by critical incidents, workplace stressors, and OSIs. PSP in higher-ranking positions may experience OSIs because of compounding critical incidents, potentially morally challenging decision making, and increased responsibilities that include the well-being of their staff: *“I know that the higher you go up, the lonelier it gets. But that’s truly what I’m feeling…I wear a white shirt, and they all wear black shirts. So there’s quite that separation prior to and there’s always that relationship gap…I truly am alone”.* P18

**Table 7:** Support Outside the RP.

Sub-Theme	Description and Supporting Quotes
Support of a Clinical Team	Support from registered healthcare providers through the provincial healthcare system, workers compensation organization, or private practice was noted as an important source of support for participants who had experienced OSIs. Engagement with clinical mental health supports assisted participants in learning skills and strategies for not only processing PPIEs but also practicing self-care and coping through the lens of maintenance and long-term well-being. Many participants spoke of the importance of ongoing support and continuing with clinical interventions initiated during the RP with a mental health clinician: *“It’s like getting a massage when you wear a duty belt, right? Like, you need one once a month, like, I’m going to go back to my other lady to deal with the day to day stuff, life, like, you know, but then I’m going to keep [psychologist] as like my work and stresses…where [psychologist] is like, a game changer for work performance, almost like, right, like, when you’re younger and playing hockey, and you have the sports performance control come in”.* P23
Support of Colleagues	Many participants reported the support they received from their peers and colleagues outside the RP was significant to their recovery and workplace reintegration. The sensitive and confidential nature of their work also contributed to a reluctance to expose those outside the PSP contexts to the harsh realities of their work. Supportive colleagues allowed for a safe place to discuss workplace stressors without concerns about secondary traumatization or breaching confidentiality: *“My supervisors and, like, the colleagues that are, like, my co-workers, who I’m very close with, have very much had a positive, like, came in, saw me, like, hey, if I can do anything to make the transition back to work easier, like let me know, type of thing. And it’s yeah, it’s been, it’s been positive”.* P10 Some participants found that the level of support and concern from others became disruptive at times even though their colleagues were well-meaning: *“I really appreciated everyone reaching out and meant a lot to me. But it almost got to a point where I was like, Hey, this is enough. Like I get it like, I’m fine. Please just leave me alone. And I didn’t want to say that because I didn’t mean it like that. But there’s just so many phone calls and so many texts”.* P20
Support of the Family	Support from family members was noted as a facilitator for mental health recovery and workplace reintegration: *“And everybody knows, because it’s been on the news so much, how the healthcare system and you especially in emergency, the emergency department, and paramedicine is kind of on the front end of everything. So everybody really understood. And were very, very supportive and asked if I needed anything, and yeah, so I had really good support*”. P7 Not all participants, however, felt their family members had the capacity to offer appropriate support. Some participants reported being worried about secondarily traumatizing family members with recollections of workplace events, which prevented them from seeking support from them. Participants who had family members such as spouses who were also PSP felt they were more likely to seek support from them opposed to non-PSP family members: *“They have very limited understanding of what I do first of all, and second of all, they have very limited understanding of how the brain works and emotion and all that stuff. So honestly, I think my [family members] probably thought I was lazy being off work. My [family member] is a social worker and works with high risk youth. So she knows and she’s taken like her leaves as well when stuff piles up too much”.* P3 Some participants noted that their family had the ability to access support through an employee assistance program, which may provide information on how to support PSP who are experiencing an OSI. It was noted, however, that participants did not think their family members had reached out to this resource. Participants suggested that family members of PSP need more education and support from the PSP organizations after a critical incident to help them better understand what their PSP family member may be experiencing: *“I was surprised too when the psychologist said, yeah, we want you to bring your spouse down too, because so many times when stuff like this happens, they’re kind of left out of the equation”.* P9
Support of the Organization	Participants noted organizational support as either a barrier or facilitator to recovery. Trust of the organization was noted by participants as critical for returning to work, with lack of trust towards the organization negatively impacting recovery from OSIs. PSP expressed the belief that the PSP organization has responsibility for the well-being of its employees given the potentially injurious nature of their work: *“I think these organizations have to be accountable for the damage that’s happening to your members”.* P23 The RP was seen as a bridge between the employee and the employer in fulfilling this responsibility. PSP reported that the amount of support received from their supervisors and organizations had an impact, either positive or negative, on their mental health recovery and workplace reintegration: *“…the local support was…was fantastic. We had our union reps both times. On the day of the incident, shortly after they contacted me both times. The RCMP has a chaplaincy program. So they reached out to me as well. And just the, the camaraderie, I guess, in the detachment. Everybody’s, you know, we’re all on the same team. So… I guess the support, in that aspect, was phenomenal”.* P9

## Data Availability

Due to the sensitive nature of the data collected for this study, data are not available for sharing.
